# Home-use servo-ventilation therapy in chronic pain patients with central sleep apnea: initial and 3-month follow-up

**DOI:** 10.1007/s11325-015-1161-7

**Published:** 2015-03-27

**Authors:** Colin M. Shapiro, Sharon A. Chung, Paul E. Wylie, Naheed K. Hossain, Rolf H. O. Holle, Russell P. Rosenberg, Mark J. Muehlbach, Robert C. Doekel, G. Vernon Pegram, Jeffrey G. Jasko

**Affiliations:** 1 Department of Psychiatry, Toronto Western Hospital, University Health Network, 399 Bathurst Street, 7 Main - 429, Toronto, Ontario M5T 2S8 Canada; 2 Youthdale Treatment Centres, Toronto, Canada; 3 Arkansas Center of Sleep Medicine, Little Rock, AR USA; 4 Western Montana Clinic, Missoula, MT USA; 5 NeuroTrials Research Inc., Atlanta, GA USA; 6 Clayton Sleep Institute, St. Louis, MO USA; 7 Sleep Disorder Center of Alabama, Birmingham, AL USA; 8 Philips Respironics, Monroeville, PA USA

**Keywords:** Chronic pain, Opioids, Sleep-disordered breathing, ASV

## Abstract

**Purpose:**

Opioid treatment of non-malignant chronic pain can result in hypoxemia, hypercarbia, and central sleep apnea. The aim of this study was to determine the initial efficacy of auto servo-ventilation (ASV) and after 3 months of home use.

**Methods:**

This prospective multicenter interventional study recruited chronic pain patients prescribed ≥100 morphine equivalents for at least 4 months.

**Participants:**

Following full-night polysomnography (PSG) to confirm the presence of sleep-disordered breathing, patients were randomized to three additional full-night-attended PSGs with continuous positive airway pressure (CPAP), ASV, and servo-ventilation with an initial mandatory pressure support of 6 cm H_2_O (ASV manual PSmin 6). Following the PSGs, patients were sent home with EncoreAnywhere and ASV with or without mandatory pressure support.

**Results:**

Based on the initial PSG studies, CPAP improved but did not normalize the apnea-hypopnea index (AHI), central apnea index (CAI), or hypopnea index (HI), as all remained elevated. Clinically significant reductions were noted after just one night of ASV and ASV manual (PSmin 6). After 3 months of ASV home use, the AHI, CAI, and obstructive apnea index (OAI) were significantly reduced when compared to baseline diagnostic levels and even when compared to respiratory disturbance indices with CPAP treatment.

**Conclusions:**

Initial and home use of ASV for 3 months resulted in significantly lower AHI, CAI, and OAI. This reduction attests to the efficacy of ASV treatment in chronic pain patients on high doses of opioids.

## Introduction

The Institute of Medicine Report on Pain [1] recognizes chronic pain as a public health challenge affecting over 100 million Americans. A statement from the American Academy of Pain Medicine [2] supports opioid treatment for chronic non-malignant pain where medically indicated, when more conservative treatments are ineffective, and if accompanied by ongoing medical monitoring. In 2009, there were close to 80 million prescriptions for opioid analgesics in the USA, with 45.7 % of all opioid prescriptions going to individuals in the age range of 40 to 59 years old [3]. Of note, this age grouping is also associated with an increased risk of developing obstructive sleep apnea (OSA) [4].

Sleep-disordered breathing (SDB) is an all-inclusive term that includes OSA, central sleep apnea (CSA), and mixed apnea (both OSA and CSA). OSA is a condition where there is intermittent partial or complete obstruction of the upper airway during sleep that limits or prevents airflow despite continued efforts to breathe. CSA is a condition where breathing is interrupted by the lack of respiratory effort. SDB is characterized by apneas and hypopneas that result in gas exchange abnormalities (hypoxemia, hypercapnia, and hypocapnia), arousal from sleep, and excessive daytime sleepiness. Individuals with undiagnosed and untreated SDB are at greater risk of increased morbidity [5] and mortality [6]. It is estimated that a significant proportion of individuals with OSA, especially those in primary care, remain undiagnosed [7].

Opioids are prescribed to treat chronic pain in up to 90 % of pain management clinic patients [8]. Although opioids are effective in the long-term management of chronic non-malignant pain [9], opioid use in patients with chronic pain was found to be a significant, dose-dependent predictor for the emergence of CSA and ataxic breathing [10, 11]. In addition to reduced ventilatory responsiveness to hypercarbia and hypoxemia [12], opioids may reduce upper airway tone predisposing the airway to instability and collapse [13]. The decline in EMG activity in the pharyngeal dilator muscles at sleep onset is much greater in patients with OSA as compared to healthy controls [14], suggesting that long-term opioid use could pose a particularly significant risk to patients with undiagnosed OSA. Jungquist and colleagues [11] did not find an exacerbation of OSA symptoms with opioid treatment, but the findings from this lone study require replication.

It is estimated that between 30 and 90 % of patients using opioids have sleep-disordered breathing [10]. In a case study of three patients on sustained-release opioids for chronic pain, Farney and colleagues [15] reported distinct patterns of SDB including ataxic breathing, CSA, sustained hypoxemia, and prolonged obstructive hypopneas. In contrast to what is typically observed in patients with OSA, apnea duration and hypoxemia were more severe and respiratory disturbances were longer in duration in NREM sleep compared to rapid eye movement (REM) sleep.

Continuous positive airway pressure (CPAP) therapy remains to be the standard first-line and effective treatment for OSA. CPAP, when titrated to the appropriate pressure, provides a positive pressure splint to prevent airway collapse and hence prevent the obstruction of the upper airway during sleep. Even though CPAP is highly effective in treating OSA, it has been considered an insufficient therapy for CSA, central apneas and hypopneas, and even periodic breathing that arises with CPAP treatment [16]. Although there is documentation of resolution of CPAP-emergent central apneic activity in some patients, this is not the case for all [17]. Further, Cassel and colleagues [18] noted the appearance of central apneas in patients with OSA on CPAP treatment despite no such activity being present during initial titration with a CPAP device.

A number of studies have demonstrated that CPAP therapy effectively abolishes obstructive apneic events but is not as effective in treating central apneas in patients with heart failure or those on opioid medications [19, 20]. In fact, in patients taking opioids, both central and obstructive events may be present throughout the night, and in some cases, the application of CPAP treatment results in an increase in the number of central respiratory events [21].

Adaptive servo-ventilation (ASV) therapy has been shown to normalize respiration in patients with Hunter-Cheyne-Stokes respiration in conjunction with heart failure [22] and in patients with persistent CSA despite CPAP treatment [19]. CSA is a common occurrence in patients with heart failure [23], and ASV therapy has been shown to improve prognosis in this patient population [24]. ASV has also been shown to be an effective counter to treat complex sleep apnea syndrome, a situation where central events emerge during CPAP titration for treatment of OSA [25].

Conflicting results have been published about the efficacy of ASV in treating SDB associated with opioid use. In a 2008 study, ASV was unsuccessful in treating SDB in patients on chronic opioid therapy [21]. The study results demonstrated that with ASV therapy, obstructive apneas decreased, while hypopneas increased and central apneas were reduced compared to baseline, but not significantly so. The latter may have occurred as a consequence of applying inadequate settings on the ASV machines utilized in the study, as the authors reported using the default settings for the end-expiratory pressure, minimum inspiratory pressure support, maximum inspiratory pressure support, and backup respiratory rate [21]. Further, ASV algorithms were initially designed to manage Cheyne-Stokes respiration (CSR) where there is a cyclical increasing and decreasing airflow pattern. Opioids may induce a less cyclical pattern, and the ASV algorithm used in the study by Farney [21] may not have adequately responded to a more variable breathing pattern. In contrast, the study by Javaheri [20] demonstrated that ASV was effective in the treatment of SDB in five patients on chronic opioids by significantly reducing both obstructive and central apneas as compared to CPAP. Similarly, Ramar and colleagues [26] found ASV to be equally effective in treating CSA and complex sleep apnea in 108 patients with chronic heart failure as well as those on chronic opioid therapy.

The above findings are a promising indicator for the effectiveness of ASV in patients with SDB associated with chronic opioid therapy but are not without significant limitations. Information is available for the comparative effectiveness of ASV and CPAP only for the initial night of treatment. It remains unknown whether the improved efficacy of ASV over CPAP treatment is sustained throughout months of therapy. Further, only the study by Javaheri [20] investigated patients with chronic pain disorders taking >100 mEq of opioid medication, but the extremely small patient sample size in that study limits the generalizability of the findings. More importantly, the participants in all of the above studies were recruited only from those referred for diagnostic evaluation of sleep apnea. These patients are quite probably those with more severe SDB and are likely not representative of most patients taking chronic opioid medications. Therefore, the efficacy of ASV versus CPAP therapy needs to be investigated over months of treatment in a study with a sufficient sample size and comprised of patients that are representative of the population of chronic pain patients being treated with opioids.

The primary aim of this study was to determine the ability of ASV therapy to correct sleep-disordered breathing with a significant central apnea component in participants with chronic pain who are treated with opioids and are naïve to positive pressure therapy. The study was designed to determine if ASV treatment improved arterial oxygen saturation during sleep and to elucidate if there is a relation between prescribed opioid dose and SDB measures.

## Methods

The System One BiPAP® autoSV Advanced (ASV, Philips Respironics) provides non-invasive ventilation in adult patients with SDB including CSR, CSA, and OSA. The ASV modulates inspiratory positive airway pressure (IPAP) according to the patient’s breathing pattern using an internal algorithm. The IPAP levels are automatically adjusted on a breath-by-breath basis in order to help stabilize the amount of air being inhaled. The ASV also provides an auto-adjusting expiratory positive airway pressure (EPAP) feature. The auto-adjusting EPAP is controlled using the device to treat airway instability or obstruction. The auto-adjusting EPAP feature includes identification of open (presumed to be central apneas) and closed (presumed to be obstructive) airway events as a critical element of its therapeutic benefits. An automatic backup rate tracks spontaneous breathing and provides an effective backup rate of 2 to 3 breaths per min below the spontaneous rate. The auto backup rate is designed to assure a rate closer to the patient’s normal rate and avoid over-ventilating the patient.

The aim of this study was to investigate the efficacy of the ASV with mandatory pressure support (ASV manual PSmin 6) and without mandatory pressure support (ASV) as compared to CPAP, during in-laboratory studies. This study introduced two control measures to promote efficacious treatment in anticipation of the decrease in respiratory drive produced by opioids. For the first control measure, the ASV device was modified to deliver a minimum minute ventilation of approximately 5 l. The ASV manual with mandatory pressure support (the second control measure) started at a minimum pressure support of 6 cm H_2_O (ASV manual PSmin 6) and was adjusted upwards as needed. The ASV (without mandatory pressure support) was set at factory settings. The eligibility criteria for the study are listed in Table 1. After the diagnostic polysomnography (PSG), those with an apnea-hypopnea index (AHI) of ≥20 and central apnea index (CAI) ≥10 events per h of sleep or at least 25 % of total sleep time (TST) below 90 % SaO_2_ and AHI ≥10 were asked to continue to participate in the study.

**Table 1:** Participants’ selection criteria

Inclusion criteria	Exclusion criteria
Males and females, ages 21–70 Able and willing to provide written informed consent Diagnosis of chronic non-malignant pain (pain present for ≥6 months) Stable regimen of opioids (oral, transdermal, and/or intravenous) for chronic pain for at least 4 weeks prior to study participation with a prescribed opioid dose equal to at least 100 mEq of morphine per 24 h Agreement to undergo an in-lab Diagnostic polysomnography (PSG) demonstrating an AHI of at least 20 and CAI ≥10 events per h of sleep, OR at least 25 % of TST below 90 % SAO_2_ saturation and AHI ≥10 Agreement to undergo 3 full-night, in-lab PSGs on positive airway pressure therapy Agreement to undergo breathalyzer testing prior to each PSG visit Ability to provide reliable documentation of opioid medications (e.g., pharmacy records) as treatment for chronic pain for the previous 30 days Willingness to undergo urine drug screening	Participation in other interventional, sleep, or pharmaceutical-related research studies within 30 days prior to giving consent Workers with variable shift schedules Previous treatment with positive airway pressure therapy within 90 days of providing consent Participants with any conditions in which positive airway pressure is medically contraindicated BMI >40 Unwilling to wear PAP Any surgery involving the upper airway, eye, nose, sinuses, or middle ear within the last 90 days Major or poorly managed medical or psychiatric condition that would interfere with the demands of the study, the use of positive airway pressure, or the ability to complete the study Previous diagnosis of severe COPD with an FEV1 < 1 l or less than 50 % predicted Presence of elevated arterial carbon dioxide levels while awake (PaCO_2_ ≥ 50 mmHg) due to intrinsic lung disease or neuromuscular or musculoskeletal disorders Participants currently prescribed with 24-h oxygen therapy

Research ethics approval was obtained for this multisite study. The full study consisted of three phases: screening, in-laboratory treatment PSGs, and 3-month device take-home evaluation. After signing informed consent, demographic data were collected and recorded. A medical history including pain diagnoses and a detailed medication assessment, including the names, frequencies, and dosages of all current medications, were performed. This multisite study involved six sleep clinics (five in the USA and one in Canada).

Participants were instructed to medicate themselves as prescribed prior to and during the screening PSG and all subsequent PSGs. Overnight diagnostic PSGs were performed using standard parameters and were scored based on standard criteria [27] at one central location. Participants who met eligibility criteria demonstrating an AHI of at least 20 and a CAI of ≥10, an OR of at least 25 % of TST below 90 % SaO_2_, and an AHI ≥10 were enrolled in the therapy phase of the study and randomized to each of the following full-night in-lab PSG titrations: CPAP, ASV, and ASV manual (PSmin 6). All therapy PSGs were scheduled within 1 week of each other, but not on consecutive nights. The CPAP starting pressure was 4 cm H_2_O and was increased 1 cm H_2_O for obstructive events associated with snoring, apneas, and hypopneas to a maximum CPAP pressure of 15 cm H_2_O (or as determined by the physician). The settings of the ASV and ASV manual (PSmin 6) are shown in Table 2.

**Table 2:** AutoSV device settings

Settings	BiPAP autoSV without mandatory pressure support (ASV)	BiPAP autoSV with mandatory pressure support (ASV PSmin 6)
Maximum pressure (cm H_2_O)	25	25
Minimum EPAP (cm H_2_O)	4	4
Maximum EPAP (cm H_2_O)	15	15
Minimum pressure support (cm H_2_O)	0	6
Maximum pressure support (cm H_2_O)	21	21
Rate setting	Auto	Auto
Rise time	2	2
Humidification	2	2
BiFlex	Off	Off

Based on the investigator’s interpretation and review of the three centrally scored therapy PSGs at each study site, each participant was asked to take home either an ASV device or a CPAP device to use for 3 months. The device settings were determined by the investigator based on the centrally scored results. Participants were asked to return to the sleep center for follow-up visits after 1 and 3 months. All take-home devices were equipped with a modem and an SD card. The daily device data were uploaded into EncoreAnywhere™ (Philips Respironics) during the 3-month at-home portion of the study, in order to assess adherence, therapy pressures, and device-output respiratory indices.

Polysomnographic endpoints were compared between the three treatment arms: CPAP, ASV, and ASV manual (PSmin 6). Due to the asymmetric distributions of the endpoints, the nonparametric Friedman analysis of variance was used to compare the related values obtained from the three therapy PSGs. Post hoc pairwise comparisons were performed using the Wilcoxon signed-rank test, with Bonferroni correction. The daily data collected from EncoreAnywhere were averaged within each participant. To calculate an unbiased participants’ average of therapy pressure and respiratory indices, the daily values were weighted relative to each day’s device usage. The 3-month follow-up data from the ASV and ASV manual (PSmin 6) groups were merged as the respiratory variables from patients using these devices were not significantly different (see Table 5). The 3-month averages of AHI, CAI, obstructive apnea index (OAI), and hypopnea index (HI) for study participants using ASV were compared to the respective measures from the CPAP titration using the Wilcoxon signed-rank test. *p* values <0.05 were considered significant. Where applicable, data in the study are expressed as median (mean ± standard deviation).

## Results

The details of the patient flow throughout the study are found in Table 3. Randomized study participants, 19 females (56 %) and 15 males, were 50.7 ± 11.6 years old and had a BMI of 30.1 ± 5.4 kg/m^2^, and racial composition was 91 % (31) Caucasian and 9 % (3) African-American. On average, study participants were on 390.1 ± 338.1 mEq of morphine (range 100–1700 mEq). Forty-seven percent (35/74; Table 3) of the participants screened had SDB. Patient demographic data for the total study group and for those participants who completed and those who discontinued the study are shown in Table 4. Compared to treatment with CPAP (Table 5), pairwise comparisons revealed clinically significant reductions in apnea-hypopnea index (AHI), central apnea index (CAI), and OAI after just one night of ASV and ASV manual (PSmin 6) use. Improvement in respiratory variables did not differ between treatment with ASV without mandatory pressure support or ASV manual (PSmin 6) with mandatory pressure support.

**Table 3:** Participants’ flow throughout the study

	Males	Females	Total
Completed study part I: diagnostic PSG screening	31	43	74
Met the criterion AHI of at least 20 and a CAI of ≥10	10	12	22
Met the criterion of at least 25 % of TST below 90 % SaO_2_ and AHI ≥10	3	5	8
Met both criteria	2	3	5
Failed both criteria	16	23	39
Randomized for the study (fulfilled inclusion/exclusion criteria)	*15*	*19*	*34*
Completed study part 2: titration with CPAP, ASV manual (PSmin 6), and ASV auto	13	18	31
Completed study part 3: 3-month at-home treatment with ASV (auto or manual)	8	11	19^a^

^a^From the 25 who started the at-home treatment phase of the study with ASV auto or ASV manual (PSmin 6), there were 19 completers

**Table 4:** Patient demographics

	Total group (*n* = 34)	Study completers (*n* = 23)	Study discontinuers (*n* = 11)
Age (years)	50.7 ± 11.6	52.5 ± 7.9	46.9 ± 16.8
Gender distribution			
Females	28	14	9
Males	11	5	6
BMI (kg/m^2^)	30.1 ± 5.4	30.7 ± 5.0	28.9 ± 6.4
Race distribution			
Caucasian	31	20	11
African-American	3	3	0
Morphine equivalents (mEq)	390.1 ± 338.1	399.5 ± 264.4	370.3 ± 472.0
Min	100	100	100
Max	1700	812.5	1700

Major diagnoses included arthritis, back/spine/neck injury, chronic pain secondary to a medical or neurological condition, and headaches/migraines. There were no differences in the demographics between those participants who completed and those who discontinued the study (age: *p* = 0.32; BMI: *p* = 0.43; mEq: *p* = 0.85)

**Table 5:** Comparison of respiratory variables across diagnostic PSG and titration studies (CPAP, ASV, and ASV manual (PSmin 6))

	Diagnostic PSG (*N* = 31)	CPAP (*N* = 31)	ASV (*N* = 31)	ASV manual (PSmin 6) (*N* = 31)	*p* values for pairwise comparisons			
					Overall *p* value (Friedman test)	CPAP vs. ASV	CPAP vs. ASV manual (PSmin 6)	ASV vs. ASV manual (PSmin 6)
AHI	32.5 (38.8 ± 31.1)	10.1 (17.4 ± 20.1)	1.4 (4.5 ± 7.3)	2.1 (7.6 ± 16.7)	*<0.001*	*<0.001*	*0.009*	>0.99
CAI	6.4 (16.1 ± 18.8)	2.4 (8.4 ± 12.4)	0.0 (0.2 ± 0.8)	0.0 (0.2 ± 0.9)	*<0.001*	*<0.001*	*<0.001*	>0.99
OAI	1.9 (9.7 ± 15.2)	2.8 (4.5 ± 6.3)	0.0 (0.3 ± 0.5)	0.0 (0.5 ± 1.1)	*<0.001*	*<0.001*	*<0.001*	>0.99
HI	10.2 (14.8 ± 12.6)	2.8 (4.5 ± 5.1)	1.4 (4.6 ± 7.4)	3.2 (7.9 ± 16.2)	0.441	–	–	–
ODI^a^	24.2 (32.8 ± 29.2)	6.0 (15.1 ± 20.2)	1.9 (5.9 ± 8.6)	2.6 (9.5 ± 19.2)	0.161	–	–	–
Av. O_2_ saturation	93.4 (92.9 ± 3.4)	94.9 (94.6 ± 2.3)	94.6 (94.6 ± 2.6)	94.6 (94.5 ± 3.2)	0.627	–	–	–
Min. O_2_ saturation	80.5 (79.9 ± 7.8)	85.0 (85.5 ± 6.0)	85.0 (82.9 ± 16.2)	86.6 (79.7 ± 22.8)	0.991	–	–	–

Data are expressed as median (mean ± standard deviation). The above *p* values for the pairwise comparisons, using the Wilcoxon signed-rank test, have undergone Bonferroni adjustment

*HI* hypopnea index, *ODI* oxygen saturation index

^a^Defined as the number of times per hour that the oxygen saturation dropped below 4 % of baseline

Table 6 presents the PSG sleep architect variables during the four PSG studies: one diagnostic study and three titration studies that were performed in counterbalanced order. Arousal index was found to be significantly (*p* = 0.021) greater during ASV manual (PSmin 6) titration compared to the CPAP titration night. No significant differences were noted for CPAP versus ASV (*p* = 0.288) or ASV versus ASV manual (PSmin 6) (*p* > 0.99). No further differences in the other PSG variables were seen across the three treatment PSGs.

**Table 6:** Comparison of PSG sleep architectural variables across diagnostic PSG and titration study (CPAP, ASV, and ASV manual (PSmin 6)) nights

	Diagnostic PSG (*N* = 31)	CPAP (*N* = 31)	ASV (*N* = 31)	ASV manual (PSmin 6) (*N* = 31)	Overall *p* value (Friedman test)
Arousal index	20.3 (22.9 ± 13.8)	11.8 (14.9 ± 8.0)	16.6 (17.7 ± 9.4)	16.1 (19.2 ± 11.7)	*0.028* ^a^
Total sleep time (TST) (min)	407.0 (409 ± 57.8)	387.0 (386.8 ± 60.2)	376.5 (377.6 ± 85.0)	385.0 (380.4 ± 67.2)	0.798
Sleep efficiency (%)	89.1 (87.9 ± 8.3)	90.0 (86.8 ± 9.7)	87.7 (83.3 ± 13.9)	86.7 (84.9 ± 10.2)	0.419
Wake after sleep onset (min)	39.6 (47.4 ± 33.5)	37.1 (50.6 ± 40.6)	45.8 (62.3 ± 50.9)	51.2 (59.0 ± 42.1)	0.313
Stage 1 (% TST)	7.8 (11.0 ± 10.2)	5.6 (8.4 ± 6.9)	6.8 (9.2 ± 8.5)	7.3 (9.4 ± 7.0)	0.201
Stage 2 (% TST)	70.9 (73.3 ± 13.0)	78.3 (76.2 ± 12.2)	74.3 (73.2 ± 11.1)	78.1 (77.0 ± 11.2)	0.206
Stage 3/4 (% TST)	0.2 (4.3 ± 7.1)	0.8 (3.6 ± 5.8)	0.6 (5.3 ± 9.5)	0.3 (3.3 ± 5.8)	0.296
REM (% TST)	11.2 (11.4 ± 8.3)	9.3 (11.8 ± 9.1)	10.3 (12.3 ± 9.4)	8.3 (10.3 ± 8.0)	0.407
Sleep onset latency (min)	4.0 (9.0 ± 12.1)	4.6 (8.8 ± 10.5)	4.3 (12.9 ± 28.4)	4.0 (9.0 ± 13.6)	0.575

Data are expressed as median (mean ± standard deviation)

^a^Pairwise comparisons, using the Wilcoxon signed-rank test and undergoing Bonferroni adjustment, indicated that the arousal index was significantly (*p* = 0.021) greater during ASV manual (PSmin 6) titration compared to CPAP. No significant differences were noted for CPAP versus ASV manual (PSmin 6) (*p* = 0.288) or ASV versus ASV manual (PSmin 6) (*p* > 0.99)

Following the PSG phase of the study, 30 participants started the at-home phase. Per the decision of the individual site investigators, four study participants (13.3 %) went home with ASV manual (PSmin 6), and the majority (21 participants, 70 %) used ASV for 3 months at home. Data for home use of ASV and ASV manual (PSmin 6) were combined. Five participants (16.7 %) were sent home with CPAP machines. Data from these latter participants will be presented elsewhere. Among the 25 participants who started the at-home phase using ASV therapy, one study participant did not use the device and was subsequently lost to follow-up. After 3 months of ASV home use (Table 7), the AHI, CAI, and OAI were all significantly (*p* < 0.001) reduced when compared to respiratory disturbance indices during the in-laboratory CPAP titration.

**Table 7:** Comparison of respiratory disturbance indices between CPAP titration and at-home use

	3-month follow-up					
	AHI (*N* = 24)	CAI (*N* = 24)	OAI (*N* = 24)	HI (*N* = 24)	Adherence	
					All days	Days used
CPAP titration	11.3 (20.3 ± 21.8)	2.6 (10.3 ± 13.5)	3.0 (5.2 ± 6.7)	3.5 (5.3 ± 5.5)	2.4 ± 2.3 (*N* = 5)	3.7 ± 1.6 (*N* = 5)
ASV home use^a^	5.8 (8.6 ± 7.9)	0.8 (1.5 ± 2.0)	0.7 (1.4 ± 1.7)	3.8 (5.7 ± 5.3)	2.4 ± 2.3 (*N* = 25)	3.6 ± 2.1 (*N* = 24)^b^
*p* value^c^	*0.021*	*0.006*	*0.002*	0.648		

Data are expressed as median (mean ± standard deviation)

^a^The ASV auto and ASV manual (PSmin 6) data combined as the respiratory variables from patients using these devices were not significantly different (see Table 5)

^b^One patient did not use the autoSV machine

^c^Wilcoxon signed-rank test

Adherence statistics were calculated based on participants’ entire 3-month at-home period or their projected 3-month visit date (if they discontinued). The one study participant who did not use their device was included in the intention-to-treat analysis. Adherence to both ASV and CPAP treatment is shown in Table 7. Overall, the ASV devices were used for 49.8 ± 36.1 % of the days of home treatment with participants using their machines for four or more hours on 30.9 ± 32.8 % of the days (*N* = 25).

## Discussion

This study is the first to report on both the acute and 3-month impact of ASV treatment of SDB in participants with chronic pain taking 100 or more milliequivalents of opioid medication. The design of this study has overcome some of the limitations of previous studies [21, 26]. Specifically, the participants in the current study had not been previously referred for investigation of a sleep breathing disorder and were recruited from chronic pain clinics, not from sleep clinics.

A large epidemiological study estimates that the point prevalence of at least one chronic painful disorder is about 1 in five individuals [28]. Given that the majority of individuals with sleep apnea remain undiagnosed [29], the findings of this study have implications for the routine clinical screening for SDB in participants with chronic pain being treated with high-dose opioids. Given that almost half of the study participants were found to have SDB, routine screening for SDB among chronic pain patients is likely to become even more of an issue based on the 2013 statement from the American Academy of Pain Medicine supporting the use of opioids to treat chronic pain combined with reports of increased mortality rates in those taking opioid medications [30].

A previous unpublished investigation found that about one third of participants in a chronic pain clinic spent about one third of the night below 90 % SaO_2_. ASV manual with mandatory pressure support (PSmin 6) was applied to address issues with decreased respiratory drive in this patient population. For this study, ASV manual (PSmin 6) (with mandatory pressure support) had no benefit over ASV without mandatory pressure support in treating sleep-disordered breathing. ASV use during the initial titration and during the 3 months of home use resulted in significantly lowered AHI, CAI, and OAI. The low treatment adherence rate during the ASV home-use phase of this study is of concern and a limitation of this study but is in line with the low CPAP adherence rates reported in the literature [31, 32]. As study participants had not previously recognized that they had a SDB, they may not have come to terms with having a sleep disorder and may not have seen the need for the treatment of their SDB. Participants’ attitude and beliefs have been shown to greatly impact treatment adherence [33], and patient education is crucial for promoting adherence to treatment for SDB [34, 35]. As such, the implementation of ASV treatment adherence strategies, including educational sessions and distribution of educational materials, should be considered for this patient population. Anecdotally, we have noted clinically that a tailor-made short educational booklet [35] providing information about CPAP can improve compliance and a similar booklet for this population would be strongly encouraged. However, our finding that respiratory indices were significantly improved with ASV treatment despite the low adherence rates attests to the unique efficacy of ASV treatment of SDB in chronic pain participants on high doses of opioids.

A significant limitation of this study is the high dropout rate. Of the 34 participants recruited, 31 completed the in-laboratory testing, but only 19 (44 % were either dropouts or were treated with CPAP) completed the 3-month at-home treatment phase. Intention-to-treat analyses were employed in this study to help reduce the impact of the dropouts on the study data. Further, although the only significant improvement in sleep architecture with treatment was a reduction in the arousal index; the arousal index was slightly higher with ASV manual PSmin 6 when compared with CPAP treatment. Slow-wave sleep levels were extremely low on the diagnostic night and did not improve with treatment. This alteration in sleep architecture is a likely consequence of the high milliequivalents taken by the study patients and the fact that opioid drugs suppress slow-wave sleep [36]. Others have also provided evidence that opioid use decreases slow-wave sleep in both healthy adults [37] and individuals with chronic pain [38].

It should be noted that the respiratory indices for the at-home study phase were recorded using the automatic event detection algorithm in EncoreAnywhere. A previous report [39] documented a strong and significant correlation (intra-class correlation = 0.8, *p* < 0.001) for AHI and AI recorded by PSG when compared to automatic event detection of respiratory events using a positive airway pressure device. The device’s algorithm did not differentiate obstructive from central events. The correlation for the HI was significant, but much lower (intra-class correlation = 0.3, *p* < 0.001).

The vicious cycle linking chronic pain and sleep disturbances is well established [40, 41]. Treatment of SDB in participants with chronic pain and on opioid therapy may reduce morbidity and mortality as a consequence of untreated SDB. A future publication from additional data collected during this study will document whether a treatment of SDB can lead to improvements in sleep, reduce pain levels, and improve daytime sleepiness, fatigue, and poor alertness.
